# Gastrointestinal pathogens detected by a multiplex PCR panel in a tertiary care center in Riyadh, Saudi Arabia (2023–2024)

**DOI:** 10.3389/fpubh.2026.1789789

**Published:** 2026-06-03

**Authors:** Nabeel Alzahrani, Hala Alfaouri, Sameera Al Johani

**Affiliations:** 1 Department of Clinical Laboratory Sciences, College of Applied Medical Sciences, King Saud bin Abdulaziz University for Health Sciences, Riyadh, Saudi Arabia; 2 King Abdullah International Medical Research Center, Riyadh, Saudi Arabia; 3 Division of Microbiology, Department of Pathology and Laboratory Medicine, Ministry of the National Guard-Health Affairs, Riyadh, Saudi Arabia

**Keywords:** coinfection, enteric pathogens, gastrointestinal infections, multiplex PCR, Saudi Arabia, seasonality

## Abstract

**Background/objectives:**

Multiplex polymerase chain reaction (PCR) assays have transformed the diagnosis of infectious gastroenteritis by enabling rapid, simultaneous detection of multiple enteric pathogens. However, contemporary data describing pathogen distribution, age-specific patterns, co-infection, and seasonality in Saudi Arabia remain limited. This study aimed to characterize the epidemiology of gastrointestinal pathogens detected by multiplex PCR in a tertiary care center in Riyadh, Saudi Arabia.

**Methods:**

We conducted a retrospective analysis of stool specimens tested using a multiplex gastrointestinal PCR panel at King Abdulaziz Medical City, Riyadh, between January 2023 and December 2024. Demographic data, detected pathogens, co-infection status, and timing of testing were analyzed. Repeat samples from the same patient within 30 days were excluded. Descriptive analyses assessed pathogen distribution by age group and season, and multivariable logistic regression was used to identify factors independently associated with co-infection.

**Results:**

Of 9,122 stool specimens processed, 2,866 positive tests from 2,189 unique patients remained after excluding 30-day repeat samples. Positivity was 31.4%, higher in pediatrics than adult patients (36.5% vs. 28.0%, *p* < 0.001). The most frequently detected pathogens were Clostridioides difficile toxin A/B, enteropathogenic *Escherichia coli*, enteroaggregative *E. coli*, norovirus, and *Salmonella* spp. Co-infections occurred in 30.8% of positive samples, more commonly in pediatric patients. In multivariable analysis, children under 2 years had significantly higher adjusted odds of co-infection compared with adults aged 18–64 years, and source of sample was independently associated with co-infection.

**Conclusion:**

Multiplex PCR testing revealed a diverse, age-dependent spectrum of gastrointestinal pathogens with a substantial burden of co-infections. These findings highlight the importance of age-specific interpretation and careful clinical correlation of multiplex PCR results.

## Introduction

Gastrointestinal (GI) infections can lead to life-threatening diseases that affect individuals across various ages globally. According to the Global Burden of Disease Study 2021, diarrheal diseases accounted for an estimated 1.17 million deaths globally, with children under five years of age experiencing the highest burden of mortality (1). Rotavirus was identified as the leading cause of diarrheal deaths among children under five, contributing to over a third of all fatal cases, followed by *Shigella* spp. and adenovirus. Other major pathogens in this age group included Cryptosporidium spp., typical enteropathogenic *Escherichia coli* (EPEC), and Shiga toxin-producing *E. coli* (STEC). Norovirus and Cryptosporidium spp. were prominent contributors in older populations (1, 2).

Early diagnosis and timely therapeutic intervention are crucial steps in the effective management of GI infection cases. Conventional diagnostic methods have relied on culture-based stool analysis to identify pathogens. Although culture-based methods have been the gold standard, they are limited by prolonged turnaround times and poor sensitivity for viral pathogens (3). In recent years, advanced molecular diagnostic approaches, such as multiplex polymerase chain reaction (PCR), have transformed the management of GI infections, significantly increasing the specificity and sensitivity of enteropathogen detection. Multiplex PCR assays enable rapid detection of multiple pathogens simultaneously, reducing diagnostic turnaround time and improving clinical outcomes and epidemiological surveillance (4, 5).

Studies from the Middle East region consistently report rotavirus as the predominant cause of pediatric diarrhea, followed by *Salmonella* spp., *Shigella* spp., and *Giardia lamblia*. Non-typhoidal Salmonella (NTS) is frequently detected, with prevalence varying widely depending on the country (6–8). In Saudi Arabia, earlier investigations conducted before widespread implementation of PCR diagnostics consistently found rotavirus, *Salmonella* spp., *Shigella* spp., *Giardia lamblia*, adenovirus, astrovirus, and norovirus among the most frequent pathogens detected in pediatric diarrhea (8–10). Recent studies have also noted the emergence of Clostridioides difficile as an important pathogen in community-associated cases, highlighting evolving epidemiological patterns (11).

Given these shifts in epidemiology and the limitations of conventional diagnostics, there is a critical need to reassess the current landscape of enteric pathogens using sensitive and rapid molecular methods. Despite the growing adoption of multiplex PCR platforms in Saudi Arabian healthcare facilities, large scale epidemiological data using these assays remain scarce. To our knowledge, this is the first study to report comprehensive multiplex PCR based gastrointestinal pathogen data from a tertiary care center and represents the largest such analysis from Saudi Arabia to date. This study aims to characterize the epidemiological spectrum of enteropathogens detected by multiplex PCR, examining age specific distributions, coinfection patterns, and seasonal trends, and identifying factors independently associated with gastrointestinal pathogen coinfection among patients presenting to a tertiary care center in Riyadh, Saudi Arabia.

## Materials and methods

King Abdulaziz Medical City (KAMC), a 2,500-bed tertiary care facility under the Ministry of National Guard Health Affairs in Riyadh, Saudi Arabia, served as the study site for this retrospective analysis. Stool specimens were collected from patients presenting with symptoms of gastrointestinal infection between January 2023 and December 2024 and were tested using the BioFire® FilmArray® Gastrointestinal Panel (BioFire Diagnostics, Salt Lake City, UT, USA), performed on the BioFire Torch System in accordance with the manufacturer’s instructions. The BioFire GI Panel is a multiplex PCR assay designed for the detection and differentiation of nucleic acids from 22 common bacterial, viral, and parasitic pathogens associated with infectious diarrhea, including Salmonella, Shigella/Enteroinvasive *E. coli* (EIEC), Campylobacter, Clostridioides difficile, Enterotoxigenic *E. coli* (ETEC), Enteropathogenic *E. coli* (EPEC), Norovirus, Rotavirus A, Adenovirus F 40/41, Cryptosporidium spp., *Giardia lamblia*, and others. The assay integrates sample preparation, amplification, and detection in a closed system, enabling rapid and accurate pathogen identification within approximately 1 h. Published studies have demonstrated the BioFire GI Panel’s high sensitivity and specificity, with performance metrics generally exceeding 95% for most targets, and minimal cross-reactivity due to its carefully designed primers and probes (12, 13).

In this retrospective study, we reviewed laboratory records at King Abdulaziz Medical City (KAMC) and included all stool specimens tested using the multiplex gastrointestinal PCR panel between January 2023 and December 2024. In accordance with the laboratory’s specimen acceptance protocol, only unformed stool specimens corresponding to Bristol Stool Chart types 6 and 7 were accepted for testing, ensuring that all analyzed samples originated from patients with diarrheal illness. To ensure that each entry represented a distinct infection episode, repeat samples from the same patient within a 30-day period were excluded. Key variables extracted included age group, sex, detected pathogen(s), and season of testing. A total of 9,122 stool specimens were tested during the study period. The study protocol was reviewed and approved by the Institutional Review Board of the King Abdullah International Medical Research Center (KAIMRC) (Approval No: 00000205525). Data were analyzed using descriptive statistical methods. Differences in positivity rates between pediatric and adult groups were compared using the Pearson chi-square test. In addition to descriptive analyses, multivariable logistic regression was performed using IBM SPSS Statistics for Windows, version 31.0 (IBM Corp., Armonk, NY, USA), to identify independent predictors of gastrointestinal pathogen co-infection, defined as detection of more than one pathogen by multiplex PCR. Age group, sex, season, year of testing, and source of sample were included as covariates. Adjusted odds ratios with 95% confidence intervals were reported, and model fit was assessed using the Hosmer–Lemeshow test. Visualization of data was done using Microsoft Excel and Power BI (Microsoft Corporation, Redmond, WA, USA), allowing the distribution of gastrointestinal pathogens to be represented in various graphical formats, including pie charts and rainbow charts.

## Results

### Demographic characteristics

A total of 2,189 patients were included in the analysis, comprising 933 pediatric and 1,256 adult patients. The median age for the overall patient population was 25 years (IQR: 4.0–59.0), with a median age of 2 years (IQR: 1.0–7.0) among pediatric patients and 55 years (IQR: 34.0–69.0) among adults. Gender distribution was nearly balanced overall, with males representing 50.3% (1103) and females 49.6% (1086). Pediatric cases showed a slight male predominance (53.8%), whereas adult cases were slightly female dominated (52.1%).

During the study period, 9,122 stool specimens were processed at our laboratory. After excluding repeat samples from the same patient within 30 days to ensure each entry represented a distinct infection episode, the analytical dataset comprised 2,866 positive tests from 2,189 unique patients, corresponding to an average of 1.31 tests per patient. The distribution of tests per patient confirmed that the cohort was not skewed toward repeatedly tested individuals. The median number of tests per patient was 1 (IQR 1–1, range 1–20), with 83.8% (n = 1,835) of patients tested only once, 94.0% tested ≤2 times, and 98.4% tested ≤4 times; only 17 patients (0.8%) were tested more than five times. This pattern was consistent across healthcare settings, with a median of 1 test per patient in outpatient (mean 1.40, range 1–20), emergency (mean 1.54, range 1–14), and inpatient (mean 1.29, range 1–13) settings. At the patient level, 739 of 2,189 patients (33.8%) had a co-infection detected in at least one test. The positivity rate was significantly higher in pediatric samples at 36.5% (1,342 out of 3,678 samples) compared to 28.0% in adult samples (1,524 out of 5,444 samples) (χ^2^ = 74.40, *p* < 0.001). Among positive samples, the majority (69.2%) contained a single pathogen, while multiple pathogen detections were observed in 30.8% of cases. Pediatric samples showed a higher rate of multiple pathogens, with two pathogens detected in 24.1%, three pathogens in 9.2%, four pathogens in 2.0% of the samples, and rare occurrences of five or six pathogens (0.15% each). In contrast, adult samples primarily presented with a single pathogen (73.5%), and fewer cases of multiple pathogen detections (Table 1).

**Table 1:** Demographic data for overall, pediatric, and adult groups.

No. (% of patients)			
Characteristics	Overall (*n* = 2,189)	Pediatric (*n* = 933)	Adult (*n* = 1,256)
Age in years, median [IQR]	25 [4.0–59.0]	2 [1.0–7.0]	55 [34.0–69.0]
Gender			
Male (%)	1,103 (50.3)	502(53.8)	601(47.8)
Female (%)	1,086 (49.6)	431(46.2)	655 (52.1)
Positivity rate (%)			
Total number of samples tested	9,122	3,678 (40.3)	5,444 (59.7)
Positive samples	2,866 (31.4)	1,342 (36.5)	1,524 (28.0)
Negative samples	6,254 (68.6)	2,334 (63.5)	3,920 (72.0)
Mixed detection (%)			
One pathogen	1985 (69.2)	865 (64.5)	1,120 (73.5)
Two pathogens	627 (21.9)	324 (24.1)	303 (19.9)
Three pathogens	211 (7.4)	124 (9.2)	87 (5.7)
Four pathogens	40 (1.4)	27 (2.0)	13 (0.9)
Five pathogens	2 (0.07)	2 (0.15)	0 (0)
Six pathogens	3 (0.10)	2 (0.15)	1 (0.1)

IQR: Interquartile Range. After excluding repeat samples from the same patient within 30 days, the analytical dataset comprised 2,866 positive tests from 2,189 unique patients (1,342 pediatric, 1,524 adult). Total samples tested and negative samples rows reflect the pre-exclusion counts.

### Pathogen distribution

Among 4,060 individual pathogen detections across 2,866 positive samples, the most frequently detected pathogens overall were toxigenic *Clostridioides difficile* (941, 23.18%), Enteropathogenic *Escherichia coli* (EPEC) (691, 17.02%), and Enteroaggregative *E. coli* (EAEC) (455, 11.22%). These were followed by norovirus GI/GII (437, 10.76%) and *Salmonella* spp. (331, 8.15%). Other notable detections included *Campylobacter* spp. (209, 5.16%), adenovirus F40/41 (78, 1.92%), *Cryptosporidium* spp. (151, 3.72%), rotavirus A (200, 4.93%), astrovirus (93, 2.29%), and Enterotoxigenic *E. coli* (ETEC) (88, 2.23%). Rarely detected pathogens included *Cyclospora cayetanensis* (1, 0.02%), *Vibrio cholerae* (3, 0.07%), and *E. coli* O157 (3, 0.07%). In pediatric patients, the leading pathogen was *C. difficile* (421, 20.89%), followed by norovirus GI/GII (307, 15.24%) and EPEC (211, 10.47%). EAEC (196, 9.79%) and *Salmonella* spp. (196, 9.79%) were equally common, while rotavirus A accounted for 173 cases (8.63%) and adenovirus F40/41 for 65 cases (3.23%). *Cryptosporidium* spp. (57, 2.83%), ETEC (38, 1.88%), Campylobacter spp. (94, 4.67%), Shigella/Enteroinvasive *E. coli* (EIEC) (35, 1.74%), and Shiga toxin–producing *E. coli* (STEC) (27, 1.34%) were also represented. Sapovirus (30, 1.49%) and astrovirus (73, 3.62%) were detected, whereas other pathogens such as Vibrio, *V. cholerae*, and *E. coli* O157 were rare (<0.1%).

*C. difficile* was the most frequently detected pathogen (520, 25.43%), followed by EPEC (480, 23.47%) and EAEC (259, 12.67%). *Salmonella* spp. (135, 6.60%), norovirus (130, 6.36%), and ETEC (84, 4.12%) were also notable. *Campylobacter* spp. (115, 5.62%), *Cryptosporidium* spp. (94, 4.60%), and Shigella/EIEC (63, 3.08%) were detected, along with *Giardia lamblia* (18, 0.88%) and *Entamoeba histolytica* (2, 0.10%). Viral pathogens beyond norovirus included adenovirus F40/41 (13, 0.64%), astrovirus (20, 0.98%), rotavirus (3, 0.15%), and sapovirus (22, 1.08%). Less frequent bacterial detections included STEC (48, 2.35%), *Plesiomonas shigelloides* (10, 0.49%), *E. coli* O157 (3, 0.15%), *Vibrio* spp. (9, 0.44%), and *V. cholerae* (2, 0.10%). Given the known high frequency of asymptomatic *Clostridioides difficile* colonization in young children, a sensitivity analysis was performed excluding children under 2 years of age from the pediatric subgroup. After exclusion, the relative contribution of *C. difficile* decreased from 20.89 to 15.21%, representing an absolute reduction of 5.68 percentage points. Despite this decrease, *C. difficile* remained among the most frequently detected pathogens in pediatric patients, while the relative contribution of other enteric pathogens, including norovirus, enteropathogenic *Escherichia coli*, enteroaggregative *E. coli*, and *Salmonella* spp., increased modestly (Supplementary Table S1).

**Figure 1:**
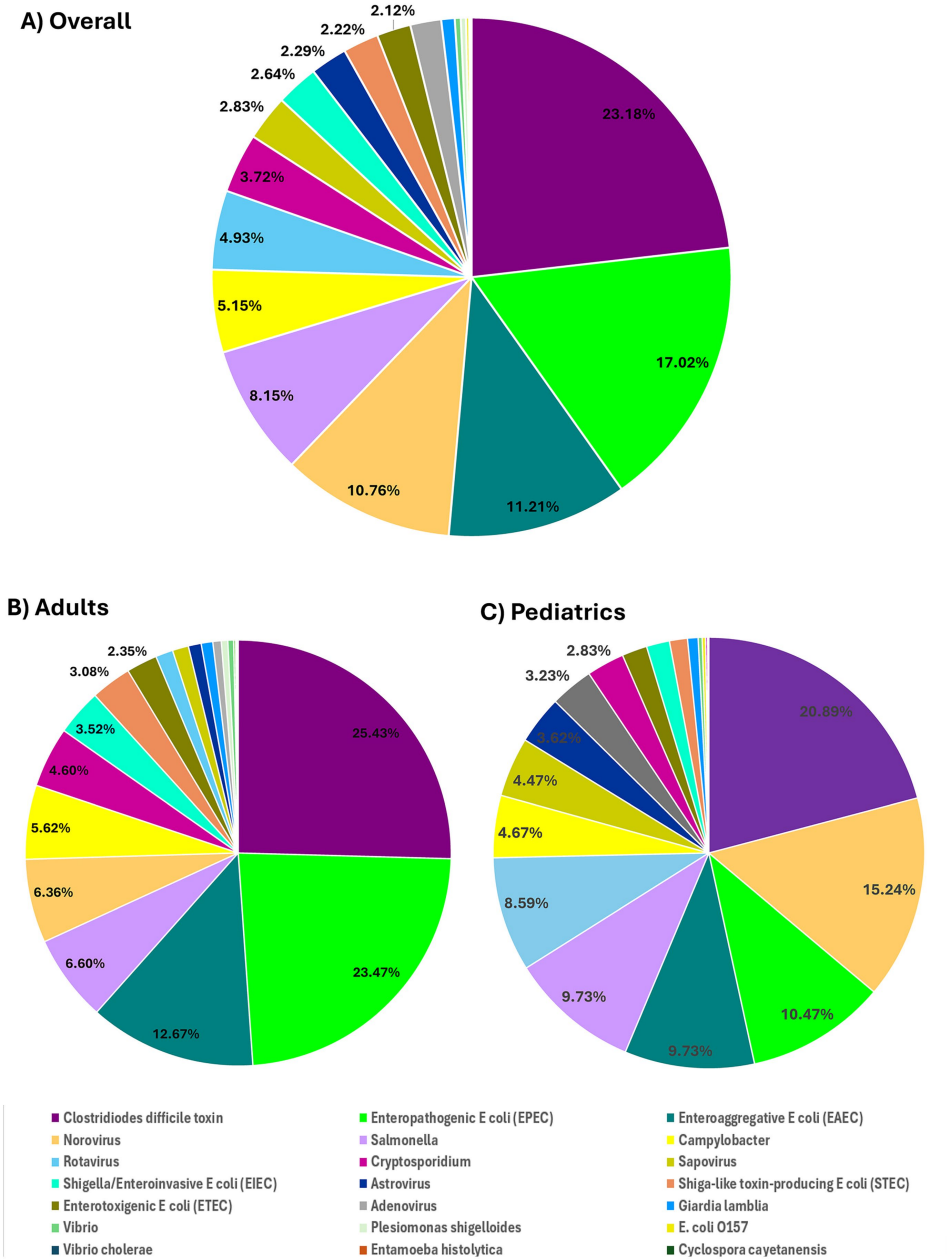
Pathogen distribution by multiplex PCR in diarrheal stool specimens: **(A)** overall cohort, **(B)** adults 18 years and older, **(C)** pediatrics under 18 years.

Expectedly, Norovirus and rotavirus were more common in pediatric patients compared to adults (15.24% vs. 6.36 and 8.63% vs. 0.15%, respectively), while bacterial pathogens such as EPEC (23.47% vs. 10.47%), EAEC (12.67% vs. 9.79%), and STEC (2.35% vs. 1.34%) were more prevalent in adults. *Giardia lamblia* detection was similar between groups, while *Campylobacter* spp. and *Cryptosporidium* spp. prevalence varied slightly, with the former marginally higher in adults and the latter more evenly distributed (Figure 1).

#### Pathogen distribution by age subgroups

**Figure 2:**
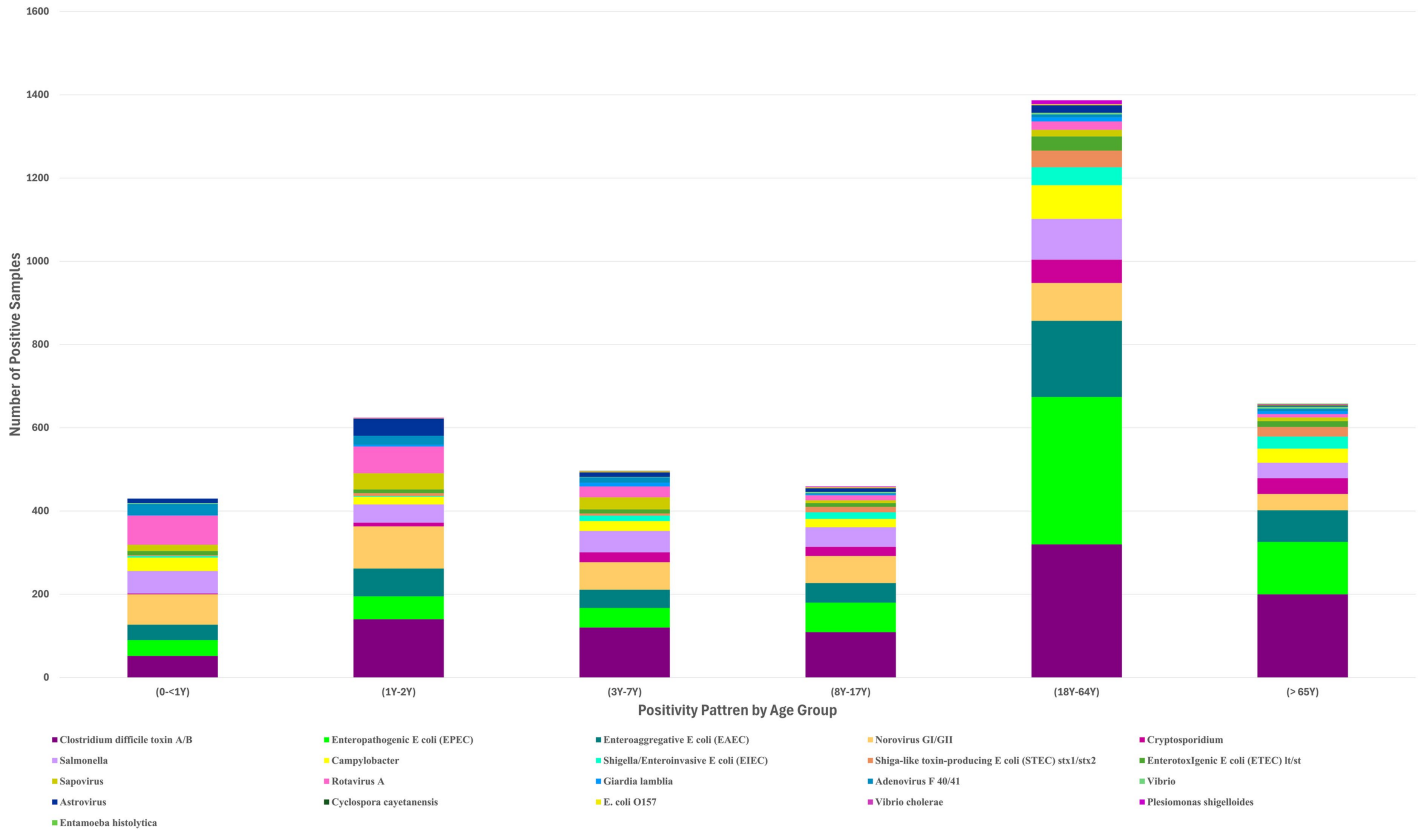
Age-specific distribution of gastrointestinal pathogens detected by multiplex PCR. Stacked bars show pathogen counts across six age groups, with each color representing a distinct bacterial, viral, or parasitic agent.

Pathogen distributions varied substantially across age groups, demonstrating a clear age-dependent shift in etiologic patterns (Figure 2). In infants younger than one-year, viral pathogens predominated, with norovirus and rotavirus representing the most frequently detected agents, alongside notable detection of *Salmonella* spp. and *Clostridioides difficile* toxin A/B. Diarrheagenic *Escherichia coli* pathotypes, particularly EPEC and EAEC, were also commonly identified, whereas protozoal pathogens were rare. In children aged 1–2 years, *C. difficile* toxin A/B emerged as the leading detected pathogen, while norovirus and rotavirus remained prominent, reflecting a mixed viral and bacterial profile and an increasing contribution of bacterial enteropathogens compared with infancy. Among children aged 3–7 years, *C. difficile* continued to dominate detections, followed by norovirus and *Salmonella* spp., with sustained representation of diarrheagenic *E. coli* and a marked decline in rotavirus detection, indicating a transition toward bacterial predominance. In children and adolescents aged 8–17 years, bacterial pathogens predominated, with *C. difficile*, EPEC, and EAEC accounting for most detections, while norovirus remained the principal viral agent and other viral pathogens were infrequently identified. Among adults aged 18–64 years, the pathogen spectrum shifted strongly toward bacterial enteropathogens, led by EPEC and *C. difficile* toxin A/B, followed by EAEC and *Salmonella* spp., with relatively lower viral detection and increased identification of Shigella/EIEC and Shiga toxin-producing *E. coli* compared with pediatric populations. In adults aged 65 years and older, *C. difficile* toxin A/B accounted for the largest proportion of detections, with persistent representation of diarrheagenic *E. coli* and norovirus, and an overall lower contribution of viral pathogens compared with younger age groups.

### Seasonal trends

Monthly analysis of positive gastrointestinal pathogen detections demonstrated clear seasonal fluctuations in both 2023 and 2024, with distinct timing and intensity of peaks between the 2 years.

In 2023, positivity rates began at relatively low levels in January and February, followed by a gradual rise through March and April. A sharper increase was noted from May through August, with the first major peak occurring in September. After a slight dip in October, a secondary surge was observed in November and December. This pattern indicates a pronounced late-summer to early-winter transmission window, with September marking the year’s highest point.

In contrast, 2024 displayed higher baseline activity from the start of the year, beginning with 150 cases in January and 180 in February. March and April maintained elevated levels, followed by a steady climb in May and June. A pronounced mid-year escalation was observed in July and August, with sustained high counts extending through September, October, and November. The highest monthly total was recorded in December, showing a protracted period of elevated positivity spanning more than half the year.

Pathogen-specific trends aligned with known seasonal patterns: Norovirus and rotavirus A peaked during the cooler months of late autumn and winter, with increases most evident between October and February. *Salmonella* spp. and ETEC demonstrated strong summer peaks, particularly from June to August, consistent with foodborne transmission amplification in warmer temperatures. *Clostridioides difficile* maintained high detection rates throughout the year, with subtle rises in autumn. EAEC, EPEC, and *Giardia lamblia* displayed relatively stable distributions, with modest increases in late summer and early autumn.

**Figure 3:**
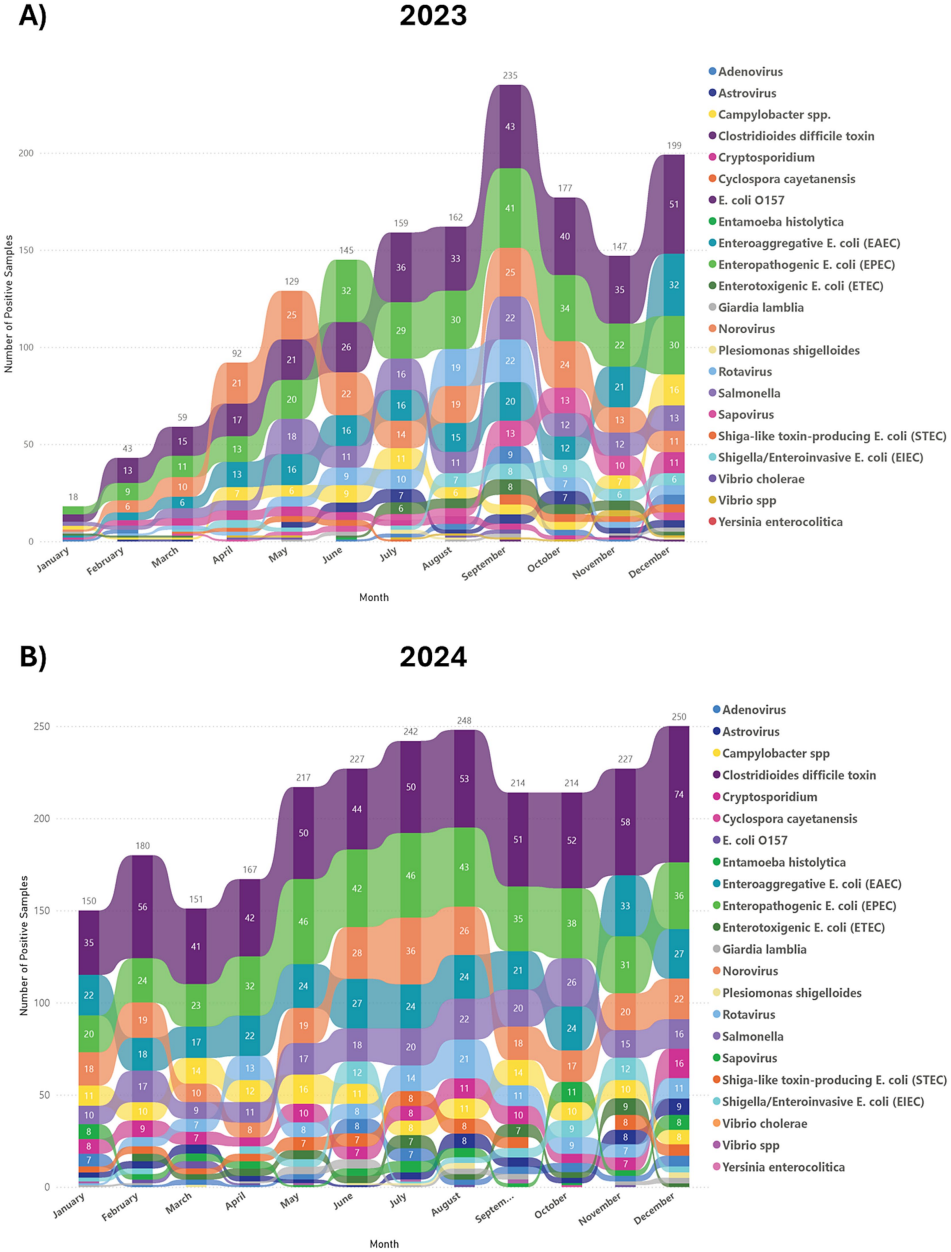
Seasonal distribution of gastrointestinal pathogens detected by multiplex PCR (2023–2024). **(A)** Monthly pathogen counts for 2023. **(B)** Monthly pathogen counts for 2024. Each color represents a distinct bacterial, viral, or parasitic agent. Testing volume increased during the early months of 2023 as multiplex PCR testing was progressively adopted at our institution; Supplementary Table S2 reports monthly testing volumes for context.

Overall, 2023 exhibited a more defined bimodal seasonal pattern with two discrete peaks, whereas 2024 was characterized by sustained high activity from mid-year onwards, suggesting either broader ongoing transmission or differences in testing and healthcare-seeking behaviors (Figure 3).

### Predictors of gastrointestinal pathogen co-infection

To identify factors independently associated with the detection of multiple gastrointestinal pathogens, a multivariable logistic regression analysis was performed with co-infection as the outcome variable (Table 2). After adjustment for age group, sex, season, year of testing, and source of sample, both age and healthcare setting were independently associated with co-infection. Compared with adults aged 18–64 years, children under 1 year (aOR 1.66, 95% CI 1.26–2.20) and those aged 1–2 years (aOR 2.13, 95% CI 1.66–2.73) had significantly higher odds of co-infection, while older pediatric and older-adult (≥65 years) groups did not differ significantly. Source of sample was also a significant predictor (*p* < 0.001), with emergency department patients showing higher odds (aOR 1.75, 95% CI 1.05–2.92) and inpatients showing lower odds (aOR 0.74, 95% CI 0.57–0.96) compared with outpatients, indicating that care setting influences the likelihood of detecting multiple pathogens. Season showed a modest association, with higher odds observed in spring and summer, while sex was not independently associated. Co-infection was less frequent in 2024 compared with 2023 (aOR 0.76, 95% CI 0.64–0.90).

**Table 2:** Multivariable logistic regression analysis of factors associated with gastrointestinal pathogen co-infection.

Variable	Adjusted OR	95% CI	*p*-value
Age group (reference: 18–64 years)			
<1 year	1.66	1.26–2.20	<0.001
1–2 years	2.13	1.66–2.73	<0.001
3–7 years	1.25	0.96–1.64	0.105
8–17 years	1.00	0.75–1.32	0.982
≥65 years	0.91	0.71–1.17	0.461
Sex (reference: female)			
Male	1.16	0.98–1.36	0.079
Season (reference: winter)			
Spring	1.33	1.02–1.72	0.033
Summer	1.36	1.06–1.75	0.015
Fall	1.13	0.87–1.45	0.362
Year (reference: 2023)			
2024	0.76	0.64–0.90	0.001
Source of sample (reference: outpatient)			
Emergency Department	1.75	1.05–2.92	0.032
Inpatient	0.74	0.57–0.96	0.025

OR, odds ratio; CI, confidence interval. Co-infection was defined as detection of more than one gastrointestinal pathogen by multiplex PCR. Variables entered into the model were age group, sex, season, year of testing, and source of sample.

## Discussion

In this two-year retrospective analysis of multiplex PCR testing for gastrointestinal infections at a tertiary care center in Riyadh, we observed a diverse and age dependent spectrum of enteric pathogens, a substantial burden of pathogen codetection, and distinct temporal patterns. Across 2,866 positive tests from 2,189 unique patients (derived from 9,122 specimens processed during the study period), the overall positivity was 31.4%, with higher yield in children (36.5%) and frequent mixed detections in pediatric samples. These findings highlight both the diagnostic utility and interpretive complexity of multiplex PCR assays in routine clinical practice and align with multi setting experiences reporting higher panel positivity in pediatric populations (4, 5, 14).

One of the most prominent observations was the high frequency of *Clostridioides difficile* toxin gene detection across all age groups, particularly among adults and older patients. While this likely reflects true infection in a subset of cases, especially in hospitalized adults, it also underscores the well-recognized limitation of highly sensitive molecular assays in distinguishing colonization from clinically significant disease. Asymptomatic colonization with *C. difficile* is common, particularly in infants and young children, where detection of toxin genes does not necessarily indicate causality for diarrheal symptoms (15, 16). Current guidelines from the Infectious Diseases Society of America (IDSA) and Society for Healthcare Epidemiology of America (SHEA) recommend against routine testing for *C. difficile* in children younger than 1–2 years unless alternative etiologies have been excluded (17, 18). These findings reinforce the importance of careful clinical correlation and diagnostic stewardship when interpreting *C. difficile* PCR results, particularly in pediatric populations. In a sensitivity analysis excluding children under 2 years of age, the relative contribution of *C. difficile* in the pediatric cohort decreased from 20.89 to 15.21%, supporting the likelihood that a proportion of detections in younger children reflects colonization rather than true infection. However, *C. difficile* remained among the most frequently detected pathogens even after exclusion, suggesting that its burden in pediatric patients should not be dismissed entirely, but rather interpreted cautiously in an age-specific clinical context (Supplementary Table S1).

Clear age-related shifts in pathogen distribution were observed, with viral pathogens, particularly norovirus and rotavirus A, predominating in infancy and early childhood, followed by a progressive transition toward bacterial enteropathogens in older children and adults. This pattern is consistent with known epidemiologic trends and reflects differences in immune maturation, environmental exposure, and the impact of vaccination programs (19, 20). The increasing predominance of diarrheagenic *Escherichia coli* pathotypes (EPEC, EAEC), *Salmonella* spp., and *Campylobacter* spp. in adolescents and adults likely reflects cumulative foodborne and community exposures. Similar age stratified patterns have been reported in pediatric cohorts using comparable syndromic panels, with EPEC, *C. difficile*, and norovirus among the most common detections (4, 5). These age specific patterns emphasize the importance of contextual interpretation and may inform empiric management strategies across different patient populations.

The high rate of pathogen codetection observed in this study, 30.8% of positive samples overall and 35.5% in pediatrics, highlights a central interpretive challenge associated with multiplex PCR panels. Detection of multiple organisms does not necessarily imply that all identified pathogens contribute to the clinical syndrome, particularly in settings where prolonged shedding and asymptomatic carriage are common (13, 14). Coinfections were more frequent in pediatric samples on descriptive analysis, which may reflect higher viral circulation, immature gut microbiota, or lower thresholds for testing in this population. However, in the multivariable logistic regression analysis, which included age, sex, season, year, and source of sample as a proxy for healthcare setting, a more nuanced relationship was observed. Children under 2 years of age demonstrated significantly higher odds of co-infection compared with adults aged 18–64 years, while older pediatric and older-adult (≥65 years) groups did not differ significantly. Source of sample was independently associated with co-infection, with emergency department patients showing higher odds and inpatients showing lower odds compared with outpatients, suggesting that healthcare setting influences the likelihood of detecting multiple pathogens. Notably, inclusion of source in the model did not substantially alter the age-related odds ratios, suggesting that healthcare setting is unlikely to be a major confounder of the relationship between age and co-infection. Exploratory stratified analyses by source indicated that the pattern of higher co-infection rates in younger children was consistent across healthcare settings. These findings suggest an age-related predisposition to pathogen co-detection, particularly among children under 2 years, although the cross-sectional design of this study precludes definitive causal inference. The lower coinfection odds in 2024 compared with 2023 (aOR 0.76, 95% CI 0.64–0.90) may reflect evolving institutional testing practices, changes in clinician ordering behavior, refinement of testing indications over the two-year study period, or shifts in circulating pathogen dynamics. These findings have important implications for antimicrobial stewardship. Rapid identification of viral etiologies in children supports supportive care and early cessation of unnecessary antibiotics, while confirmed *C. difficile* in an adult with compatible symptoms supports targeted treatment and prompt isolation. In a Taiwanese hospital using the same multiplex platform, availability of panel results was associated with significant reductions in overall antimicrobial use and higher rates of targeted therapy for *C. difficile* in adults compared with children, consistent with age specific differences in colonization and disease probability (5). Our findings support similar stewardship opportunities in our setting, though prospective studies linking panel results to therapeutic decisions and clinical outcomes would strengthen these inferences.

Distinct seasonal patterns in pathogen detection were observed, with viral pathogens more frequently detected during cooler months and bacterial enteropathogens demonstrating relative increases during warmer periods. These trends are consistent with established epidemiologic patterns of viral gastroenteritis and foodborne bacterial infections reported globally and regionally (19, 21). A Danish region wide implementation of syndromic testing similarly demonstrated viral peaks in late winter and bacterial peaks in summer (22). Regionally, a recent syndromic PCR-based study from Al-Ahsa, Saudi Arabia, reported concordant seasonality patterns, with summer peaks for diarrheagenic *E. coli* pathotypes and *C. difficile*, and winter predominance of *Campylobacter* and *Cryptosporidium* (23). In Qatar, Abdel-Rahman et al. demonstrated that viral pathogens, particularly norovirus and rotavirus, predominated among pediatric AGE cases, with seasonal peaks during cooler months, consistent with the patterns observed in our cohort (24). The persistent year-round detection of *C. difficile* likely reflects healthcare associated transmission dynamics and testing practices within tertiary care settings rather than true seasonal variation. The concordance of our findings with known seasonal trends supports the external validity of this dataset and the value of continued local molecular surveillance to anticipate healthcare resource needs.

This study has several limitations. First, the analysis was conducted at a single tertiary care center, with the majority of samples (87.9%) originating from hospitalized patients, which may limit generalizability to community or primary care settings. Data on whether these patients were admitted primarily for gastrointestinal illness or tested incidentally during hospitalization for unrelated conditions were not available, and therefore the clinical context of testing could not be determined. Second, multiplex PCR detects pathogen nucleic acids and does not distinguish between active infection and asymptomatic carriage, particularly for organisms such as *C. difficile* and certain diarrheagenic *E. coli* pathotypes. Third, clinical data regarding symptom severity, antimicrobial exposure, and patient outcomes were not systematically available, precluding correlation between molecular findings and clinical disease attribution or assessment of stewardship impact. Fourth, rotavirus vaccination history was not available for the study population, which limits interpretation of rotavirus A detections, particularly in pediatric patients, as it was not possible to distinguish between infections in unvaccinated children and potential vaccine breakthrough cases. Fifth, we did not perform confirmatory culture or molecular subtyping, which limits characterization of antimicrobial susceptibility and outbreak linkage. Sixth, testing volume increased during the initial months of 2023 as multiplex PCR was progressively integrated into routine clinical practice, which may affect the comparability of absolute monthly detection counts during that period. Finally, although repeat samples within 30 days were excluded to reduce duplication, reinfection or prolonged shedding beyond this interval cannot be fully excluded. These constraints are common in laboratory based observational studies and can be addressed by prospective designs that link diagnostics with clinical, stewardship, and economic endpoints (4, 5, 22).

In conclusion, multiplex PCR testing revealed a diverse and age dependent spectrum of gastrointestinal pathogens in a large tertiary care population in Saudi Arabia, with a substantial burden of pathogen codetection and distinct seasonal trends. Viral pathogens predominated in younger age groups while bacterial pathogens, particularly toxigenic *C. difficile* and diarrheagenic *E. coli*, were prominent in adults. The high frequency of *C. difficile* detection and coinfections underscores the need for age aware interpretation and diagnostic stewardship when using multiplex molecular panels. Ongoing local surveillance combined with clinical correlation should further improve patient care and inform public health responses to gastrointestinal infections in the region.

## Data Availability

The datasets analyzed in this study are not publicly available because they are governed by the data governance policies of the King Abdullah International Medical Research Center (KAIMRC). The data are available from the corresponding author upon reasonable request and subject to approval by KAIMRC.
