# Supplementary material for: Gastrointestinal pathogens detected by a multiplex PCR panel in a tertiary care center in Riyadh, Saudi Arabia (2023–2024)
Source: Front Public Health. 2026 Jun 3;14:1789789. doi: 10.3389/fpubh.2026.1789789 (PMC13272143; doi:10.3389/fpubh.2026.1789789)
Supplement: Supplementary file 1 [file Supplementary_File_1.DOCX]

**Supplementary Table S1. Pediatric pathogen distribution before and after exclusion of children under 2 years of age from the pediatric subgroup.**

| Pathogen | Pediatric cohort, all ages <18 years n (%) | Excluding children <2 years n (%) | Absolute change, percentage points |
| --- | --- | --- | --- |
| *Clostridioides difficile* toxin A/B | **421 (20.89)** | **286 (15.21)** | **-5.68** |
| Adenovirus F40/41 | **65 (3.23)** | **65 (3.46)** | **+0.23** |
| Enteropathogenic *Escherichia coli* (EPEC) | **211 (10.47)** | **211 (11.22)** | **+0.75** |
| Cryptosporidium spp. | **57 (2.83)** | **57 (3.03)** | **+0.20** |
| Enterotoxigenic *E. coli* (ETEC) lt/st | **38 (1.89)** | **38 (2.02)** | **+0.14** |
| Norovirus GI/GII | **307 (15.24)** | **307 (16.33)** | **+1.09** |
| Campylobacter spp. | **94 (4.67)** | **94 (5.00)** | **+0.33** |
| Enteroaggregative *E. coli* (EAEC) | **196 (9.73)** | **196 (10.43)** | **+0.70** |
| Rotavirus A | **173 (8.59)** | **173 (9.20)** | **+0.62** |
| *Salmonella* spp. | **196 (9.73)** | **196 (10.43)** | **+0.70** |
| Shiga toxin-producing *E. coli* (STEC) stx1/stx2 | **27 (1.34)** | **27 (1.44)** | **+0.10** |
| Sapovirus | **90 (4.47)** | **90 (4.79)** | **+0.32** |
| *Shigella*/Enteroinvasive *E. coli* (EIEC) | **35 (1.74)** | **35 (1.86)** | **+0.12** |
| Astrovirus | **73 (3.62)** | **73 (3.88)** | **+0.26** |
| *Giardia lamblia* | **16 (0.79)** | **16 (0.85)** | **+0.06** |
| *Entamoeba histolytica* | **1 (0.05)** | **1 (0.05)** | **0.00** |
| *Vibrio* spp. | **6 (0.30)** | **6 (0.32)** | **+0.02** |
| *Vibrio cholerae* | **1 (0.05)** | **1 (0.05)** | **0.00** |
| *E. coli* O157 | **5 (0.25)** | **5 (0.27)** | **+0.02** |
| *Plesiomonas shigelloides* | **3 (0.15)** | **3 (0.16)** | **+0.01** |

**Data are presented as number of detections and percentage of all pediatric pathogen detections within the specified subgroup. Children under 2 years of age were excluded in the sensitivity analysis because of the known high frequency of asymptomatic Clostridioides difficile colonization in this age group. Absolute change is expressed in percentage points.**

**Supplementary Table S2. Monthly distribution of samples tested and positive detections, 2023–2024.**

| Month | Total samples tested (2023) | Total samples tested (2024) | Positive samples (2023) | Positive samples (2024) |
| --- | --- | --- | --- | --- |
| January | 75 | 396 | 10 | 119 |
| February | 112 | 400 | 32 | 130 |
| March | 126 | 369 | 42 | 113 |
| April | 181 | 417 | 63 | 119 |
| May | 246 | 505 | 85 | 152 |
| June | 261 | 522 | 96 | 160 |
| July | 308 | 541 | 102 | 175 |
| August | 342 | 562 | 114 | 179 |
| September | 422 | 512 | 146 | 159 |
| October | 382 | 510 | 120 | 151 |
| November | 336 | 533 | 104 | 164 |
| December | 467 | 597 | 143 | 188 |
| Total | **3258** | **5864** | **1057** | **1809** |

***Note: All figures reflect the number of specimens processed per month before exclusion of repeat samples within 30 days. The analytical dataset after repeat-sample exclusion comprised 2,866 positive tests from 2,189 unique patients. Testing volume increased during the early months of 2023 as multiplex PCR testing was progressively adopted at our institution before stabilizing.***
